# Subcutaneous Nephrovesical Bypass in Kidney Transplanted Patients

**Published:** 2010-08-01

**Authors:** M. Yazdani, M. R. Gharaati, M. Zargham

**Affiliations:** 1 *Department of Urology and Renal Transplantation, Isfahan University of Medical Sciences, Isfahan, Iran, *; 2 *Isfahan Kidney Disease Research Center, Department of Urology and Renal Transplantation, Isfahan University of Medical Sciences, Isfahan, Iran*

**Keywords:** Ureteral obstruction, Nephrovesical bypass, Kidney transplantation

## Abstract

Background: Renal transplant ureteral stricture or obstruction is a rare but devastating complication after renal transplantation.

Objective: To determine the efficacy and complications of subcutaneous prosthetic ureters as a salvage procedure in transplanted kidneys with recurrent ureteral obstruction.

Methods: 5 subcutaneous prosthetic ureters were inserted in 5 kidney recipients who had recurrent ureteral stenosis and failed endoscopic and open reconstructive surgeries. The prosthetic ureter consisted of an internal silicone tube covered by a coiled PTFE tube. The proximal end of the tube was introduced in the transplanted kidney percutaneously, the tube was passed through a subcutaneous tunnel, and the distal end was inserted in the bladder through a small suprapubic incision.

Results: The mean follow-up of patients was 11.3 months. One of the patients re-operated two days after the procedure because of urinary leakage from the distal end of the prosthetic ureter. No infection or tube encrustation was encountered.

Conclusion: Subcutaneous prosthetic ureter is a safe alternative for permanent percutaneous nephrostomy in transplanted kidneys with obstructed ureter and failed endoscopic and open procedures.

## INTRODUCTION

Renal transplant ureteral stricture or obstruction is a rare but devastating complication after renal transplantation. In most centers retrograde or antegrade endourologic procedures with ureteral dilatation and insertion of a ureteral stent is the first intervention. If endoscopic managements fail, the second choice is usually open surgery for ureteroneocystostomy, pyelovesicostomy, or pyeloureteral anastomosis to the native ureter. In rare cases, both of the endoscopic and open procedures fail to treat the obstruction and further surgeries are often impossible or unsuccessful because of excessive fibrosis in the operation site and impaired vascularity of the transplanted ureter. Under this condition, there is no choice other than placing a percutaneous nephrostomy tube in the transplanted kidney. But, long-term percutaneous nephrostomy has major disadvantages such as recurrent infections, encrustation, obstruction, displacement and dislodgement which need regular change of the catheter. Most importantely, long-term percutaneous nephrostomy is associated with a significant decrease in the quality of life [1,2].

In such cases, subcutaneous prosthetic ureters may be suitable alternatives to further open and endoscopic surgeries. These artificial ureters have been used for end-stage patients with impassable ureteral obstruction caused by pelvic malignancies and there are very limited experiences with using these stents in transplanted kidneys.

Herein, we present our experience with using subcutaneous prosthetic ureters in kidney transplanted patients with ureteral stricture who had failed endoscopic and open procedures.

## PATIENTS AND METHODS

Between November, 2008 and Febrary, 2010, we implanted five prosthetic ureters in five kidney transplant patients (three men and two women) with ureteral stricture. The mean age of patients was 50.3 (range: 7-61) years. All patients had a history of failed endoscopic and at least one open reconstructive surgery to repair the obstructed ureter and had nephrostomy tubes inserted in the transplanted kidney at the time of surgery. Urine culture was negative in all patients pre-operatively and bladder capacity and function was normal.

**Figure 1:**
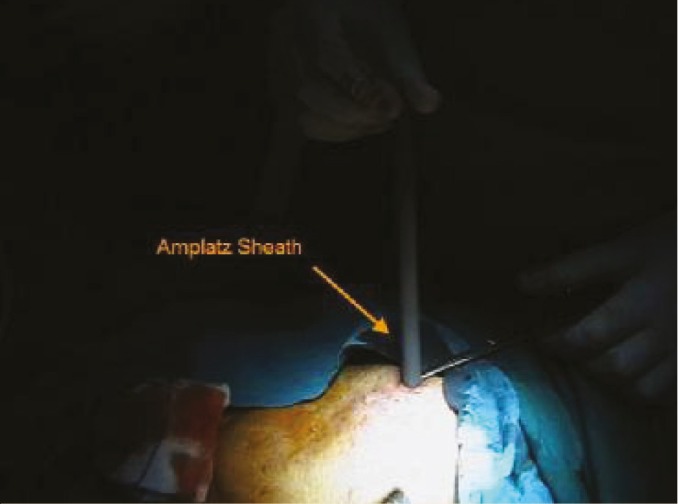
Amplatz sheath is introduced into the transplanted kidney under fluoroscopic guidance

A Detour double tube system was used as nephrovesical bypass. It consists of two co-axial tubes: a porous 27F polytetrafluoro-ethylene (PTFE) outer tube and an inner 17F silicone tube. The procedure was done under general anesthesia in four and spinal anesthesia in one of the patients. Patient was in the supine position. The previously existed nephrostomy tract was used for placing the proximal end of bypass tube in two patients and a new tract was created in other three patients, because the nephrostomy tract was too lateral and kinking of the bypass tube was probable. The tract was then dilated serially with metal dilatators to 30F and an amplatz sheath was introduced under fluoroscopic guidance (Fig. 1).

**Figure 2:**
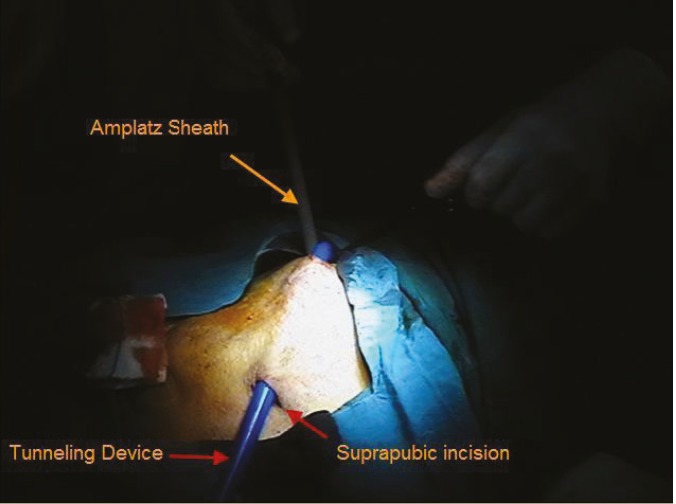
Subcutaneous tract is created between the amplatz site and suprapubic region using a tunneling device

**Figure 3:**
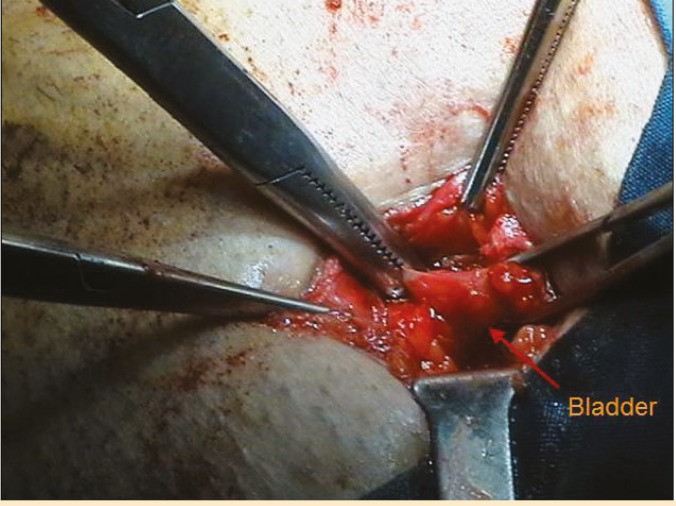
A small area of bladder dome is exposed through the suprapubic incision

A 2-cm incision was created in the suprapubic region to access the bladder. A tunneling device was used to create subcutaneous tract between amplatz site and suprapubic incision (Fig. 2). Then, the bypass tube was introduced into the amplatz sheath and proximal end of silicone tube was positioned in the calyx of the transplanted kidney so that the radio-opaque ring marker was placed at the junction of calyx and renal parenchyma. The amplatz sheath was removed. The distal end of bypass tube was then passed through the tunneling device to the suprapubic incision and the tunneling device was removed too. The length of bypass tube was adjusted for each patient, the excess length was cut and the outer PTFE tube is peeled away for 2–3 cm at the distal end to expose the inner silicone tube. The bladder was distended via a Foley catheter and a small region of bladder dome was exposed (Fig. 3). The distal end of inner silicone tube was fenestrated and then introduced into the dome of bladder via a small incision. The outer PTFE layer was fixed to bladder serosa using 4-0 absorbable sutures.

The Foley catheter was left for 7–10 days and then removed. Prophylactic antibiotic was administered from 24 hours before surgery until the day of Foley catheter removal. All patients received their usual immunosuppressive regimen before and after surgery.

## RESULTS

**Figure 4:**
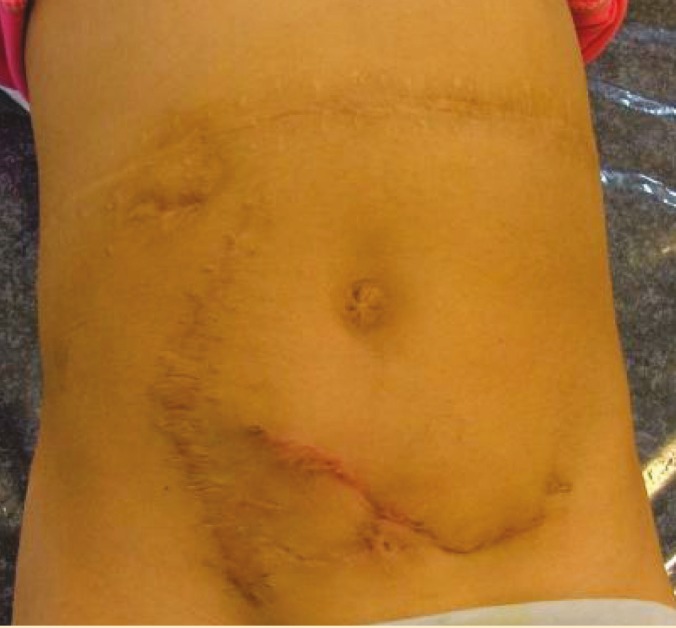
Multiple scars on the abdomen of a 7-year-old kidney transplanted girl as the result of prior open surgeries

The youngest patient was a 7-year-old girl with a history of right and then left nephrectomy and chemotherapy in infancy because of bilateral Wilm’s tumor. She was transplanted one year before and had a history of two failed open surgeries aimed at reconstructing the renal transplant ureter. She had multiple scars on her abdomen and suprapubic region (Fig. 4). The mean follow-up of patients was 11.3 (range: 4-15) months.

One of the patients had excess urinary leak from suprapubic incision post-operatively and needed a second surgery to fix the problem. Leakage was the result of tube kinking at the distal end. Besides this patient, there was no other immediate complication in these five patients.

Late complication occurred in one patient in the form of prolonged frequency and urgency. Symptoms responded to bladder specific anticholinergic (Tolterodine) but not completely. Asymptomatic bacteriuria was encountered in one patient. He had episodes of bacteriuria before the surgery and it was not a new onset complication. No cases of tube obstruction or infection were encountered in our series.

## DISCUSSION

In 1960’s and early 1970’s, the first attempts were taken place to bypass the ureteral obstruction with silicone prostheses [3]. Thereafter, there are reports about using artificial ureters in patients with end-stage malignancies or benign diseases and ureteral obstruction in whom endoscopic procedures or open surgery could not resolve the stricture or reconstruct the ureter [1,2,4-6].

There are rare case reports or case series about using these artificial ureters in the renal transplanted patients with complicated ureteral obstruction. Desgrandchamps, *et al*., reported one of the first cases in this regard [7]. They used a subcutaneous nephrovesical diversion for total replacement of the ureter in one renal transplant patient. Follow-up period was eight months and no complication was occurred. In 1998, Desgrandchamps, *et al*. reported urinary diversion with artificial ureters in three cases of ureteral necrosis after renal transplantation [8]. With the mean follow-up of 2.5 years, no late complications were observed and all grafts had good function post-operatively. Reflux was present in all three patients but without any clinical manifestation. In this study, they used subcutaneous nephrovesical bypass in patients who had failed endoscopic management and as an alternative to open surgery. They did not tried open surgery because of its potential hazard to the graft. We used the artificial ureter when both the endoscopic and open managements were failed and we believe that prosthetic ureter is the last choice in patients with a long life expectancy such as kidney transplanted patients. Andonian, *et al*., implanted artificial ureters in two renal transplanted patients after failed endourologic or open management of ureteral obstruction [9]. After 12 and 15 months of follow-up, the renal function was stable with no evidence of obstruction. They recommended that long-term follow-up is needed to evaluate the rate of encrustation and obstruction.

Infection of implants is a major concern in renal transplanted patients. Jabbour, *et al*., reported a series of 35 subcutaneous prosthetic ureters implanted in 27 patients with malignant or benign etiologies for ureteral obstruction [10]. Four of those patients had ureteral necrosis after kidney transplantation. In the long-term follow-up, no infection or encrustation of the silicone inner tube occurred in patients and long-term complications were rare—even in the case of immunosuppressed patients with transplanted kidneys. In our patients, infection was not a problem during the follow-up period.

## CONCLUSION

Subcutaneous prosthetic ureter is a safe, highly effective and minimally invasive alternative for permanent percutaneous nephrostomy in transplanted kidneys with failed endoscopic and open procedures.
